# Barriers to and facilitators of rehabilitation services for people with physical disabilities: A systematic review

**DOI:** 10.4102/ajod.v2i1.22

**Published:** 2013-09-19

**Authors:** Nondwe B. Mlenzana, Jose M. Frantz, Anthea J. Rhoda, Arne H. Eide

**Affiliations:** 1 Faculty of Community and Health Sciences, University of the Western Cape, South Africa; 2 SINTEF Technology and Society, Oslo, Norway; 3 Extraordinary Professor, Centre for Rehabilitation Studies, Stellenbosch University, South Africa

## Abstract

**Background:**

As health care practitioners, it is important to have an understanding of the common barriers to and facilitators of the rehabilitation services we provide.

**Objectives:**

This article aimed to review the relevant literature regarding barriers to and facilitators of rehabilitation services for people with disabilities.

**Method:**

Articles for the period 1990–2010 using descriptors related to rehabilitation services, barriers, facilitators and the physically disabled population were retrieved for this review.

**Results:**

A total of 19 article titles were identified from references of other articles but following application of the inclusion criteria selected for this review, only six articles were chosen. Five of these articles were qualitative studies and one was a quantitative study. Barriers and facilitators regarding rehabilitation services highlighted by participants in the studies included a perception that health professionals have a lack of understanding of rehabilitation for people with disabilities and there was a lack of information sharing from health professionals about the rehabilitation process. On the other hand some participants reported that health professionals demonstrated confidence in the disability and rehabilitation process during consultation and highlighted that their needs were met by the rehabilitation professionals.

**Conclusion:**

Even though there were few studies highlighting the barriers to and facilitators of rehabilitation services, they highlighted that there are gaps in the process of rehabilitation services provided. It would be advisable for health professionals to take cognisance of the issues highlighted in this study in order to make rehabilitation services more effective.

## Introduction

According to the recently launched *World report on disability* (World Health Organization [WHO] 2011), 15% of the population globally presents with disabilities, with physical disability being most prevalent. The numbers of disabled people are increasing globally due to population growth, ageing, emergence of chronic diseases and medical advances that preserve and prolong life (WHO 2005). These trends create overwhelming demands for health and rehabilitation services, which are very far from being met, particularly in low-income countries (WHO 2005).

Disability is conceptualised as a complex process involving bodily functions, health, environment, activity limitations and restrictions in social participation (WHO 2001). Optimal health care for people with physical disabilities is essential if their quality of life is to improve. Understanding the needs of the physically disabled population may be a complex process as it involves understanding the person, the society in which he or she lives, and how these interact. Various studies done in the period 1991–1997 state that people with disabilities as a group have challenges with regard to access to health services (Davis & O’Brien 1996; Gold *et al.* 1997; Weissman *et al.* 1991). In order to assist in improving the health outcomes of people with disabilities, it is essential to understand what the barriers to and facilitators of this population are as it relates to medical services that include rehabilitation. Hence this article aimed to review the relevant literature regarding barriers and facilitators with regard to rehabilitation services for people with disabilities.

There are different categories of people who are in need of rehabilitation services. In the rehabilitation centres in the Western Cape Province, the following common conditions are seen: arthritis, spinal cord injury, head injury, neuromuscular disorders, stroke, fractures and amputations (Metro District Health Services [MDHS] 2009). Investing in health and rehabilitation services contributes not only to ensuring equality of opportunities and good quality of life for people with disabilities, but also to promoting social participation and valuable contribution to society. The United Nations’ *Convention on the rights of persons with disabilities* (United Nations [UN] 2006) underlines the rights of individuals with disabilities to play an active role in society, and that accessing rehabilitation services plays a key role in achieving this.

More than a decade ago, Keith (1998) highlighted that there is a need to understand the patient’s view on service delivery and explore whether rehabilitation services acknowledge patient perspectives and make relevant adjustments. This was supported by Haynes, Devereaux and Guyatt (2002), who highlighted the role of patient preferences in disease management and considered it important that their views are heard. In a more recent study, Van Til *et al.* (2010) highlighted the need to understand the barriers that patients experience in the field of rehabilitation and how these can be overcome. The recommendation is to involve clients in decision making regarding their rehabilitation so that clients can be part of the process. They further recommended that studies need to be conducted to explore the barriers to rehabilitation and how to overcome them.

A client-centred and holistic philosophy takes into account the goals and expectations of the client and should consider the individual’s broader life circumstances (Cott 2004). Cott (2004) suggested the following important components of client-centred rehabilitation:

… individualization of programs to the needs of the client for a smooth transition between rehabilitation programs and the community; sharing of information and education that is appropriate, timely, and according to clients’ wishes; family and peer involvement in the rehabilitation process; coordination and continuity within and across sectors; and outcomes that are meaningful to the client. (p. 1418)

This is also in line with the primary health care (PHC) approach to health in South Africa, which highlights that ‘specific rehabilitative services should include a basic assessment of people with disabilities, followed by an appropriate treatment programme, in consultation with the disabled person and his family’ (Department of Health 2000:43).

Rehabilitation services in PHC settings are important for the welfare of patients with physical disabilities. Over the last decade new rehabilitation evidence for specific interventions has been conceptualised but not practiced (Wade & De Jong 2000). Studies that were chosen for this systematic review focused on clients with disabilities who received rehabilitation services at community level. PHC rehabilitation professionals offer non-pharmacological interventions that have both a preventive and therapeutic role in the management of patients with physical disabilities. However, there is a need to identify explicit service-delivery models that operationalise a PHC and rehabilitation approach to patients with physical disabilities. Understanding the views of key stakeholders relating to current services will assist in identifying the gaps in the rehabilitation services being offered. During this literature review, no previously published systematic reviews on this specific topic could be found. Hence, this study aims to explore literature on rehabilitation services for people with physical disabilities, in order to identify the barriers to and facilitators of accessing such services.

## Methodology

A systematic approach to the review was adopted and it is reported in a narrative form. The protocol to develop a systematic review was done as a guideline before the study was conducted. This systematic review is one of the objectives of a bigger project (Project number: 10/1/23).

### Criteria for the review

Articles considered for inclusion could be either qualitative or quantitative studies and had to be published in English (as interpretation of the studies would be easier for researchers) during the period 1990–2010. To be included in the review the studies had to focus on people with physical disabilities that attended rehabilitation services and were exposed to rehabilitation services, either institution based or community based.

### Search strategy

The search strategy was implemented as follows. Databases such as CINAHL with full text, ERIC, Academic search premier, MEDLINE, Health resource-consumer edition, Health source: Nursing/Academic edition, PsychARTICLES, SocIndex with full text and Ebscohost were searched for this review. The terms used to search for literature included rehabilitation service, facilitators and barriers, physically disabled, rehabilitation service providers and user satisfaction. Search terms such as positives and negatives, people with disabilities, physical therapists, occupational therapists, doctors, nurses, social workers, and client satisfaction were used alternatively to search terms such as barriers and facilitators. References used in other studies were also perused to identify articles that did not emerge in the initial database search. Studies were excluded if they did not specifically focus on rehabilitation services. Six articles meeting the criteria were found through the identified databases, and based on their titles, 19 articles were identified from the reference lists of these articles.

### Methods of review

Initially, the search was conducted by one researcher, and the abstracts and titles were screened by two reviewers. Documents for the last two decades were reviewed by applying the PIO method (Moyer 2008) – where ‘P’ stands for population, ‘I’ stands for intervention of interest and ‘O’ stands for outcome – to the abstract. The PIO method was used to select articles relevant for the study. The population included patients with physical disabilities, the intervention was access to rehabilitation services and the outcome focused on barriers or facilitators. This method assisted with identification of participants used in the studies, barriers to and facilitators of rehabilitation services and outcomes of these studies. If articles did not meet the criteria they were excluded from the study. Full-text articles were obtained once they were included following the PIO process. Inclusion into the systematic review was also based on the methodological quality of the study.

The reviewers used a critical review form (Potvin 2007) for the quantitative studies (see Table 1) and a critical appraisal skills programme (CASP) form (see Table 2) to assess the methodological quality of the qualitative studies (Critical Appraisal Skills Programme [CASP] 2004).

**TABLE 1:** Critical review form questions.

Number	Questions
1	Was the purpose stated?
2	Was relevant background literature reviewed?
3	Describe design.
4	Was sample size justified?
5	Specify the frequency of outcome measurement.
6	Was intervention described in detail?
	Was contamination avoided?
7	Were results reported in terms of statistical significance?
	Was the analysis appropriate for the type of outcome measures and the methodology?
	Was clinical importance reported?
	Were drop-outs reported?
8	Were the conclusions made by the authors appropriate given the study method and results?

**TABLE 2:** Critical Appraisal Skills Programme (CASP) review questions.

Number	Questions
1	Was there a clear statement of the aims of the research?
2	Is the qualitative methodology appropriate?
3	Was the research design appropriate to address the aims of the research?
4	Was the recruitment strategy appropriate to the aims of the research?
5	Were the data collected in a way that addressed the research issue?
6	Has the relationship between researcher and participants been adequately considered?
7	Have ethical issues been taken into consideration?
8	Was the data analysis sufficiently rigorous?
9	Is there a clear statement of findings?
10	How valuable is the research?

**FIGURE 1:**
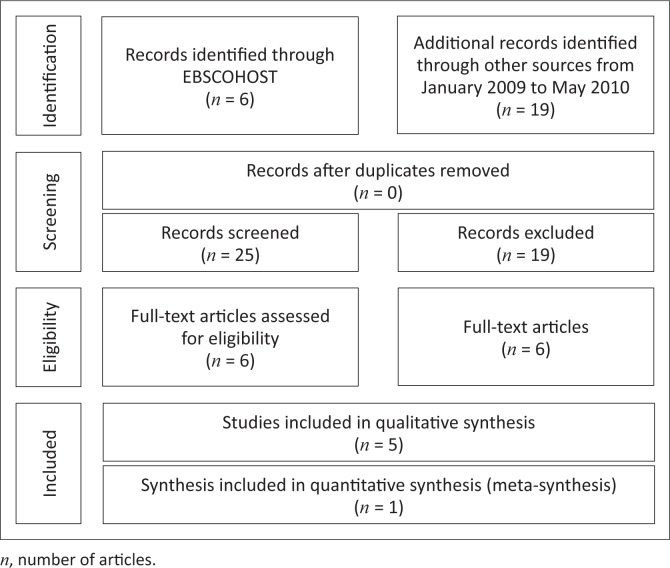
Search strategy followed for articles used in this study (Moher *et al.* 2009).

Only 6 out of the 25 article titles identified during the literature review met the inclusion criteria. The omitted articles (19) were mostly excluded as they did not address the aim of this review or did not include the identified population based on the CASP form. A flowchart of the process followed to identify the articles used in this study is presented in Figure 1.

### Data extraction

A self-developed data extraction form was used to extract the data from the studies, using criteria that were determined prior to the data extraction. The data extraction form was designed to extract information such as author, date of publication, country, population including sample size, gender, educational level and type of disability (see Table 3). Out of the 25 articles the reviewers identified 6 articles that met the criteria for the review. Reviewers compared opinions and reached consensus on the final articles to be included.

**TABLE 3:** Articles that were reviewed and met the criteria of the study.

Number	Author	Gender	Race	Country	Type of study	Occupation	Level of education	Study population
1	Mangset *et al.* (2008)	Females: 7 Males: 5	Not mentioned	Norway	Qualitative	Pensioners	Not mentioned	Stroke patients
2	Zongjie *et al.* (2007)	Females: 175 Males: 285	Han and other nations	China	Quantitative	Officials and leaders: 51 Professional technical personnel: 41 Clerks: 24 Shop workers: 51 Factory workers: 159 Military: 12 Others: 144 Retired: 226	College and above: 51 Middle school: 337 Primary school: 49 Illiterate: 23	Stroke, spinal cord injuries, cerebral palsy, head injuries
3	Vincent *et al.* (2007)	Females: 7 Males: 10	Not mentioned	Canada	Qualitative	Not mentioned	Elementary: 9 Secondary: 6 Post-secondary: 2	Stroke
4	Kroll *et al.* (2006)	Females: 16 Males: 20	White, Black, Asian and Hispanic	USA	Qualitative	Not mentioned	Not mentioned	Spinal cord injury, stroke, multiple sclerosis
5	Crisp (2000)	Females: 21 Males: 14	Not mentioned	Australia	Qualitative	Employed: 7 Unemployed: 24	Students: 3	Physical disability
6	Williams and Bowie (1993)	Not specified, 181 participants	Not mentioned	UK	Qualitative	Not mentioned	Not mentioned	Severely physically disabled

### Conceptual framework for analysis

The World Health Organization’s (2001) International Classification of Functioning, Disability, and Health (ICF) introduced a model that promotes an understanding of the complexity of health, and well-being practices are an indication of this. The ICF provides a framework for viewing activity limitation and participation restrictions from three broad and different perspectives, which include physiologic, physical–environmental and psychosocial functions. When evaluating the current articles, the authors took the ICF into consideration. The two main components of the ICF are firstly the individual’s functioning and disability, and secondly contextual factors. The main aspect important for this review is activities and participation from an individual perspective. According to the ICF, contextual factors include the environmental factors which comprise the physical, social and attitudinal environment in which people live and conduct their lives. Personal factors include an individual’s life and living, and comprise features of the individual (gender, race, age, health conditions, fitness, lifestyle, coping styles, social background, education, profession etc.). The ICF provided a useful framework and vocabulary for identifying the barriers and facilitators for this review. This framework was thus used to analyse the information reflected in the studies according to the various themes or categories that emerged. Based on these themes linked to participants’ view of barriers and facilitators, the authors attempted to create a comprehensive picture.

## Results

The findings of the review focused on the barriers and facilitators regarding rehabilitation services identified by physically disabled people. Five of the six studies were from developed countries (Australia, Norway, Canada and England) and one from a developing country (China). These studies are reported on individually based on the aim of the study, population and outcome of the study. Contextual factors include environmental and personal factors. Environmental factors are classified as the physical, social or attitudinal world and may be facilitating or hindering. Personal factors may include socio-demographic data (age, education, profession, race and gender), co-morbidities, fitness, lifestyle, coping mechanisms and psychological status. The studies included focused on different aspects of personal factors. Williams and Bowie (1993) focused on the personal factors with regards to the disorder and thus the need for specific health professionals to focus on their disability. Zongjie *et al.* (2007) focused on personal factors such as finances, years of disability and the patients understanding of rehabilitation services. On the other hand, Vincent *et al.* (2007) focused on personal factors such as behaviour, language and sexual relations.

In addition, with regard to the contextual factors, barriers to as well as facilitators of rehabilitation services are found in the physical, social and attitudinal environment in which people live and conduct their lives. Mangset *et al.* (2008) used semi-structured interviews that aimed to explore patient satisfaction as a quality indicator in the area of elderly stroke patients’ rehabilitation. The population were clients who had a stroke and were between the ages of 60 and 87 years. In this study, participants identified the following *facilitators* related to the rehabilitation process: being treated with humanity by health professionals, being acknowledged as individuals, having their autonomy respected, having confidence and trust in health professionals, and exchange of information.

Williams and Bowie (1993) used interviews in order to report on the quality of monitoring and management of the needs of severely physically disabled residents who were in regular contact with health professionals. The population were clients with severe physical disability between the ages of 16 and 64 years. Based on this study’s findings, identified *barriers* to rehabilitation included that their needs were not met by health professionals in terms of activities of daily living, lack of communication, lack of resources in the areas of psychology, speech therapy and neuropsychology, lack of education given to the disabled, and lack of community awareness regarding disability.

Zongjie *et al.* (2007) used a series of comprehensive questionnaires to explore the requirements of disabled residents regarding rehabilitation services. The population were clients with disabilities between the ages of 30 and 70 years. The *facilitators* identified by the participants in this study included provision of information, doctors having good skills, easy access to doctors, good understanding of rehabilitation services, confidence in the value of rehabilitation services, and easily accessible rehabilitation services.

Vincent *et al.* (2007) used focus group discussions and explored partially met and unmet rehabilitation needs of older adults who had suffered a stroke and who lived in the community. The population were clients with stroke over the age of 65 years. These clients identified *barriers* to rehabilitation as being that rehabilitation was not personalised to the needs of the patient and there was not enough support for patients.

Kroll *et al.* (2006) used focus group discussions to explore barriers and strategies affecting the utilisation of primary preventive services for people with physical disabilities. The population were clients 18 years and older with a physically disabling condition. Clients identified the following structural-environmental and procedural *barriers*: poor facilities, equipment and procedural accessibility issues, poor transportation, poor appointment scheduling, poor patient-provider communication, professional manner, disability-specific knowledge, personal motivation, cognitive issues, information and self-education, and lack of a personal doctor or usual source of care.

Lastly, Crisp (2000) used interviews in order to examine the perceptions of people with disabilities concerning their interaction with health and rehabilitation professionals. The population were clients with disabilities between 24 and 56 years. *Barriers* to rehabilitation included the following: ineffective health and rehabilitation professionals, family members as part of rehabilitation process were devaluing the clients, rehabilitation was associated with unwanted dependency and social discomfort, and dissatisfaction with the help received. *Facilitators* included meaningful assistance from health and rehabilitation professionals, having therapeutic relationships with health and rehabilitation professionals, and being assertive and independent in rehabilitation.

The services utilised by participants included rehabilitation medical services, psychological services and social services. Rehabilitation education was provided as part of the rehabilitation process.

## Discussion

The aim of the current study was to explore the literature on rehabilitation services for people with physical disabilities, in order to identify the barriers to and facilitators of accessing such services. Within the context of the ICF it is important to consider various factors that influence an individual’s reason to access health services, such as policies, individual and contextual factors.

### Policies

The PHC approach includes five types of care, namely promotive, preventive, curative, rehabilitative and palliative. Within this approach health care must be accessible, affordable, appropriate and accountable. Five of the studies included in the review were from developed countries that adopt a health care system similar to the PHC approach. Table 4 highlights the various health care systems that are followed in different countries, including South Africa.

**TABLE 4:** Health care systems identified.

Country	Aim of health care system
South Africa (African National Congress [ANC] 1994)	Health care in South Africa varies from the most basic primary health care, offered free by the state, to highly specialised health services available in the both the public and private sectors. Thus parallel private and public health systems exist. The public system serves the vast majority of the population, but is underfunded and under-resourced.
Norway (Johnsen 2006)	The organisational structure of the Norwegian health care system is built on the principle of equal access to services. The emphasis in their health system is based on the primary health care model where all inhabitants should have the same opportunities to access health services, regardless of social or economic status and geographic location.
China (Xinming 2005)	The health policy in China focuses on addressing the health challenges of the 21st century and ensuring access to care. Priorities include preventive, promotive and curative care.
Canada (Irvine Ferguson & Cackett 2005)	According to Irvine *et al.* (2005), there is a need to accommodate the changing pattern of care from an institutional to a community-based model. This will allow accessibility of the health centres to all members of the country.
UK (Boyle 2011)	Health services in England are largely free. The National Health System provides preventive medicine, primary care and hospital services to all.

### Individual factors

It is evident from the studies that patients with varying conditions access rehabilitation services and that they have positive and negative experiences regarding the service. Participants’ service-related expectations were the same; they both complimented and were dissatisfied with rehabilitation service received. Although four of the six studies reported on the education level of participants, conclusions cannot be drawn to how this has influenced experience with rehabilitation services. Literature indicates, however, that receiving health information from health professionals such as physicians is an important measure for increasing knowledge amongst users of the service as they are perceived to have clear knowledge about conditions (Tian *et al.* 2011). In addition, Paasche-Orlow *et al.* (2005) highlighted that health literacy is associated with education, ethnicity and age. They further highlighted that there is a need to simplify health information provided to patients as part of health services.

### Contextual factors

One of the main outcomes of the review was that clients with physical disabilities identified health professionals’ attitude towards them as both a facilitator and a barrier. Respect and empathy were highlighted as facilitators. On the other hand, people with disabilities were concerned about health professionals who focused on their disabilities and not their health. A client-centred approach is favoured in the literature, which emphasise that clients regard respect, autonomy and acknowledgement as individuals as important aspects (Mangset *et al.* 2008). The need for client education was also identified as a barrier. This is supported by other researchers (Harris, Hayter & Allender 2008) who suggested that communication and lack of information were barriers related to health care professionals when managing patients with chronic illnesses. In the review, communication was also experienced positively and negatively at the rehabilitation centres. Some participants felt that health care professionals exchanged information during consultation whilst others felt that communication was lacking, especially regarding issues such as assistive devices and education about health conditions that people with disabilities presented with (Vincent *et al.* 2007; Williams & Bowie 1993). In addition, participants were concerned about the lack of resources in the areas of psychology, speech therapy and neuropsychology when incorporated in rehabilitation services. This was seen to limit the holistic approach to the management of a person with a disability who is in need of one of these services. Although certain types of services were found to be limited, participants in this review felt that rehabilitation services were easily accessible to them as they valued the existence of rehabilitation centres in their areas or community (Zongjie *et al.* 2007).

### Implication for practice

It is evident from the review that there are mixed emotions about rehabilitation services. This review therefore highlights for rehabilitation practitioners the gaps that need to be addressed to make this service a comprehensive one. It is also evident from the review that contextual factors play a major role in understanding the impact of disability and the need for rehabilitation services. The factors highlighted in this review allow the consideration of patient-centred service provision models to be explored further.

## Limitations of the study

This review has several limitations. Because only English language articles were included, it is possible that this review is a not complete representation of the available evidence. In addition, the databases accessed was limited to those available at a single institution and thus could present a publication bias. As both qualitative and quantitative articles were included it was difficult to compare the results of the studies.
